# Selective Laser Trabeculoplasty in Advanced-to-Severe Primary Open-Angle Glaucoma: Six-Month Clinical Outcomes

**DOI:** 10.7759/cureus.117782

**Published:** 2026-10-11

**Authors:** Leena K Awi, Zulhilmi Abdul Razak

**Affiliations:** 1 Ophthalmology, International Islamic University Malaysia, Kuantan, MYS

**Keywords:** advanced primary open-angle glaucoma, glaucoma, intraocular pressure, selective laser trabeculoplasty, visulas green

## Abstract

Introduction: Selective laser trabeculoplasty (SLT) is an established treatment for reducing intraocular pressure (IOP) in patients with primary open-angle glaucoma (POAG). However, evidence on the use of VISULAS green SLT in advanced-to-severe POAG remains limited, particularly in real-world clinical settings. This study aimed to evaluate the six-month outcomes of VISULAS green SLT in patients with advanced-to-severe POAG.

Methods: This retrospective cohort study included seven patients (10 treated eyes) with advanced-to-severe POAG who underwent SLT at a tertiary ophthalmology center in Malaysia between January and December 2025. IOP and topical IOP-lowering medication burden were assessed at baseline and at one week, one month, three months, and six months after SLT. Changes over time were analyzed using the Friedman test, followed by exploratory Wilcoxon signed-rank tests for paired comparisons. Subsequent glaucoma interventions were also recorded.

Results: Of the 10 treated eyes, seven (70%) had advanced POAG and three (30%) had severe POAG. The mean patient age was 65.29 ± 8.88 years, and five patients (71.4%) were female. Overall, mean IOP decreased from 22.40 ± 7.21 mmHg at baseline to 19.29 ± 2.81 mmHg at six months, although the number of eyes with available measurements decreased from 10 to 7. Among the seven eyes with complete six-month data, mean IOP increased from 18.86 mmHg at baseline to 19.29 mmHg at six months, representing a paired mean increase of 0.43 mmHg. No statistically significant overall change in IOP was observed (Friedman test, p = 0.511). The median topical medication burden remained at two medications throughout the follow-up (p = 0.092). None of the seven evaluable eyes (0%) achieved the predefined target IOP at six months. Subsequent glaucoma surgery was documented in three eyes (30%), comprising two trabeculectomies and one iStent implantation.

Conclusions: In this small real-world cohort, no statistically significant changes in IOP or topical medication burden were demonstrated over six months following VISULAS green SLT. These findings should be interpreted cautiously given the very small sample size and incomplete follow-up and should be considered exploratory rather than evidence of an absence of treatment effect. SLT may serve as an adjunctive or interim treatment in selected patients who decline or temporarily defer surgery; however, close monitoring and timely escalation of treatment remain essential.

## Introduction

Glaucoma is a group of progressive optic neuropathies characterized by retinal ganglion cell loss, optic nerve damage, and corresponding visual field defects [1,2]. It is a major cause of irreversible blindness worldwide. Primary open-angle glaucoma (POAG) is one of the most common forms of the disease and typically progresses slowly and asymptomatically, potentially leading to substantial visual impairment before diagnosis [3].

Intraocular pressure (IOP) is the principal modifiable risk factor for the development and progression of glaucoma [4,5]. Treatment options include topical IOP-lowering medications, laser therapy, and incisional glaucoma surgery. Although topical medications are widely prescribed, long-term treatment may be associated with poor adherence, ocular surface adverse effects, and an increased treatment burden, particularly among patients requiring multiple medications [6].

Selective laser trabeculoplasty (SLT) is a non-incisional treatment that lowers IOP by selectively applying laser energy to the pigmented trabecular meshwork while minimizing damage to surrounding structures [7]. The ZEISS VISULAS green laser system uses a 532-nm frequency-doubled solid-state laser to deliver SLT. A prospective multicenter clinical investigation demonstrated the efficacy and safety of VISULAS green SLT in lowering IOP [8].

SLT may be used as an initial treatment for POAG or ocular hypertension or as an adjunct to existing IOP-lowering therapy [9]. The Laser in Glaucoma and Ocular Hypertension (LiGHT) trial demonstrated that SLT can provide effective IOP control as a first-line treatment while reducing reliance on topical medications in appropriately selected patients [10].

Despite the established role of SLT, evidence regarding its effectiveness in advanced-to-severe POAG remains limited. Schlote and Kynigopoulos investigated SLT in patients with early and advanced open-angle glaucoma, reporting variable treatment responses and noting that some patients subsequently required filtering surgery [11]. Baseline IOP is another important factor influencing treatment response. Hodge et al. identified higher baseline IOP as a significant predictor of SLT success at one year [12]. Similarly, Pillunat et al. identified pretreatment IOP as an important predictor of IOP reduction following SLT in patients receiving maximally tolerated medical therapy [13].

Evaluating SLT in advanced-to-severe glaucoma is particularly important because patients with substantial optic nerve damage have limited remaining visual function and often require lower target IOP levels to minimize further disease progression. The Malaysian Clinical Practice Guidelines for the Management of Glaucoma emphasize that target IOP should be individualized according to disease severity, baseline IOP, risk of progression, and other patient-specific factors [4].

Understanding SLT outcomes in this population may help clarify its role as an adjunctive or interim treatment, particularly for patients who decline or temporarily defer incisional glaucoma surgery. Therefore, this study primarily aimed to evaluate changes in IOP over six months following VISULAS green SLT in patients with advanced-to-severe POAG treated within the ophthalmology services of International Islamic University Malaysia (IIUM). The secondary objectives were to assess changes in topical IOP-lowering medication burden, achievement of the predefined target IOP, and subsequent glaucoma surgical interventions.

## Materials and methods

Study design and setting

This retrospective cohort study was conducted within the ophthalmology services of International Islamic University Malaysia (IIUM), Kuantan, Pahang, Malaysia. All SLT procedures were performed at the IIUM Eye Specialist Centre by a single glaucoma surgeon using the VISULAS green 532-nm laser system. A standardized 360° SLT treatment was performed in all included eyes.

Study population and sampling

Patients aged over 40 years with advanced-to-severe POAG who underwent SLT between January and December 2025 were identified retrospectively from existing clinical records. Consecutive sampling was used to include all eligible patients treated during the study period. The final sample comprised 7 patients and 10 treated eyes.

Inclusion and exclusion criteria

Patients were eligible if they were older than 40 years, had a confirmed diagnosis of advanced-to-severe POAG based on the most recent reliable visual field examination before SLT, and underwent treatment using the VISULAS green laser system during the study period. Disease severity was classified according to the Malaysian Clinical Practice Guidelines for the Management of Glaucoma.

Patients with ocular hypertension, secondary glaucoma, other forms of glaucoma, previous glaucoma filtering surgery, or missing baseline IOP measurements were excluded. Eyes with incomplete follow-up were retained in the descriptive analyses using available observations.

Data collection and outcome measures

Clinical data were obtained retrospectively from existing patient records. Information collected included patient age, sex, laterality, glaucoma severity, baseline IOP, number of topical IOP-lowering medications, follow-up IOP measurements, and subsequent glaucoma interventions.

IOP was measured using Goldmann applanation tonometry during routine clinical visits. IOP and medication burden were evaluated at baseline, one week, one month, three months, and six months following SLT.

Topical medication burden was defined according to the number of active IOP-lowering ingredients rather than the number of bottles. Accordingly, a fixed-combination preparation containing two active ingredients was counted as two medications.

The primary outcome was the change in IOP during the six-month follow-up period. Secondary outcomes included changes in topical IOP-lowering medication burden, achievement of the predefined target IOP, and subsequent glaucoma surgical interventions.

The predefined treatment target was an IOP of 14 mmHg or lower together with a reduction of at least 30% from baseline IOP.

Potential confounding factors

Potential factors influencing treatment response included baseline IOP, glaucoma severity, previous topical medication use, medication adjustments during follow-up, bilateral treatment, and subsequent glaucoma surgery. These factors were considered when interpreting the findings but were not formally adjusted for in a multivariable model because of the small sample size. Follow-up completeness was also assessed to identify possible attrition bias.

Statistical analysis

The treated eye was the primary unit of analysis. Descriptive statistics were used to summarize demographic characteristics, IOP, and topical medication burden. Continuous variables were reported as mean ± standard deviation and median, as appropriate, while categorical variables were presented as frequencies and percentages.

Exploratory Wilcoxon signed-rank tests were performed to compare baseline IOP with each follow-up measurement using eyes with available paired observations. Mean paired differences were also calculated descriptively.

Changes in topical medication burden across baseline, one week, one month, three months, and six months were evaluated using the Friedman test.

Because some patients contributed both eyes, a patient-level sensitivity analysis was performed to account for potential correlation between fellow eyes. For patients with bilateral treatment, IOP measurements from both eyes were averaged at each time point, and longitudinal IOP changes were subsequently evaluated using the Friedman test.

Statistical significance was defined as p < 0.05.

Sample size

No formal a priori sample size calculation was performed. Given the retrospective nature of the study and the limited number of eligible patients, all eligible patients identified during the study period were included. The final sample comprised 7 patients and 10 treated eyes.

Ethics statement

This study involved a retrospective review of existing clinical records without additional patient interventions.

## Results

A total of 10 treated eyes from 7 patients with advanced-to-severe POAG were included. The mean patient age was 65.29 ± 8.88 years (range: 50-76 years). Five patients (5/7, 71.4%) were female, and two patients (2/7, 28.6%) were male. Six eyes (6/10, 60%) were left eyes, and four eyes (4/10, 40%) were right eyes. Seven eyes (7/10, 70%) were classified as having advanced glaucoma, while three eyes (3/10, 30%) had severe glaucoma (Table 1).

**Table 1:** Demographic and clinical characteristics Age and sex are reported at the patient level, whereas laterality, glaucoma severity, and six-month follow-up availability are reported at the eye level. IOP = intraocular pressure, N: number of patients or treated eyes.

Characteristics	N	%; mean ± SD
Number of patients	7	
Number of treated eyes	10	
Age, years	7	65.29 ± 8.88
Age range, years		50-76
Female	5	71.40%
Male	2	28.60%
Right eye	4	40%
Left eye	6	60%
Primary open-angle glaucoma	10	100%
Advanced glaucoma	7	70%
Severe glaucoma	3	30%
Six-month IOP data available	7	70%
Defaulted before six months	2	20%
Underwent glaucoma surgery before the six-month assessment	1	10%

IOP outcomes

The mean IOP was 22.40 ± 7.21 mmHg at baseline, 21.70 ± 8.54 mmHg at one week, 21.75 ± 7.91 mmHg at one month, 20.12 ± 6.20 mmHg at three months, and 19.29 ± 2.81 mmHg at six months. Follow-up availability decreased from 10 eyes at baseline to 7 eyes (7/10, 70%) at six months. Descriptive statistics are presented in Table 2.

**Table 2:** Descriptive statistics for IOP All IOP measurements are expressed in millimeters of mercury (mmHg). The number of available eyes varies across follow-up periods. IOP: intraocular pressure, N: number of eyes with available measurements.

Time point	N	Mean	SD	Median	Minimum	Maximum
Baseline	10	22.40	7.21	22.00	12	36
One week	10	21.70	8.54	20.00	13	44
One month	8	21.75	7.91	21.00	15	40
Three months	8	20.12	6.20	20.00	12	32
Six months	7	19.29	2.81	20.00	16	24

Among the seven eyes with complete measurements at all five time points, mean IOP was 18.86 mmHg at baseline and 19.29 mmHg at six months, representing a descriptive paired mean change of +0.43 mmHg. The complete-case IOP trend is illustrated in Figure 1.

**Figure 1:**
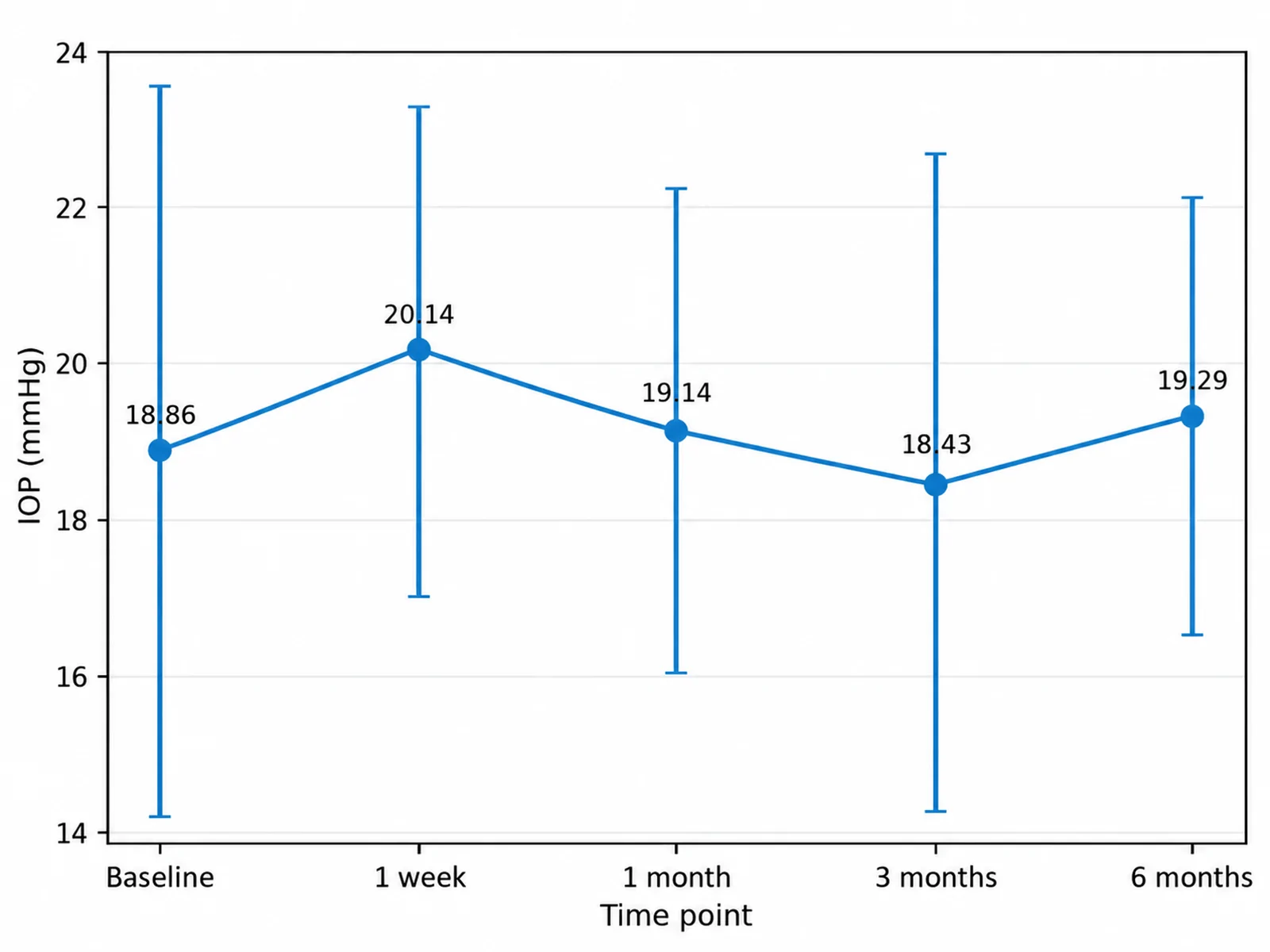
Mean IOP across five time points among complete cases Mean intraocular pressure (IOP) is expressed in millimeters of mercury (mmHg), at baseline, one week, one month, three months, and six months following selective laser trabeculoplasty among seven eyes with complete longitudinal data (N = 7). Error bars represent ±1 standard deviation (SD).

Exploratory Wilcoxon signed-rank tests comparing baseline IOP with each follow-up measurement did not demonstrate statistically significant differences. Mean paired differences were calculated descriptively and were -0.70 mmHg at one week, +0.75 mmHg at one month, -0.88 mmHg at three months, and +0.43 mmHg at six months. The corresponding Wilcoxon p-values were 1.000, 0.531, 0.406, and 0.688, respectively.

**Table 3:** Paired baseline-to-follow-up IOP comparisons All IOP measurements are expressed in millimeters of mercury (mmHg). Mean change was calculated as follow-up IOP minus baseline IOP; negative values indicate a reduction in IOP. None of the paired comparisons reached statistical significance (p ≥ 0.05). IOP: intraocular pressure, n: number of eyes with paired measurements.

Comparison	Paired n	Baseline mean	Follow-up mean	Mean change	Wilcoxon W	p-value
Baseline vs one week	10	22.40	21.70	-0.70	10.0	1.000
Baseline vs one month	8	21.00	21.75	+0.75	7.0	0.531
Baseline vs three months	8	21.00	20.12	-0.88	8.0	0.406
Baseline vs six months	7	18.86	19.29	+0.43	11.5	0.688

Antiglaucoma medication burden

The mean number of topical IOP-lowering medications was 2.40 at baseline and one week, 2.50 at one month, 2.75 at three months, and 2.57 at six months. Among the seven eyes with complete medication data, the median medication burden remained at two active IOP-lowering ingredients throughout follow-up. The Friedman test did not demonstrate a statistically significant change in medication burden across the five measurement periods (χ²(4, N = 7) = 8.000, p = 0.092; Kendall's W = 0.286). The medication trend is illustrated in Figure 2.

**Figure 2:**
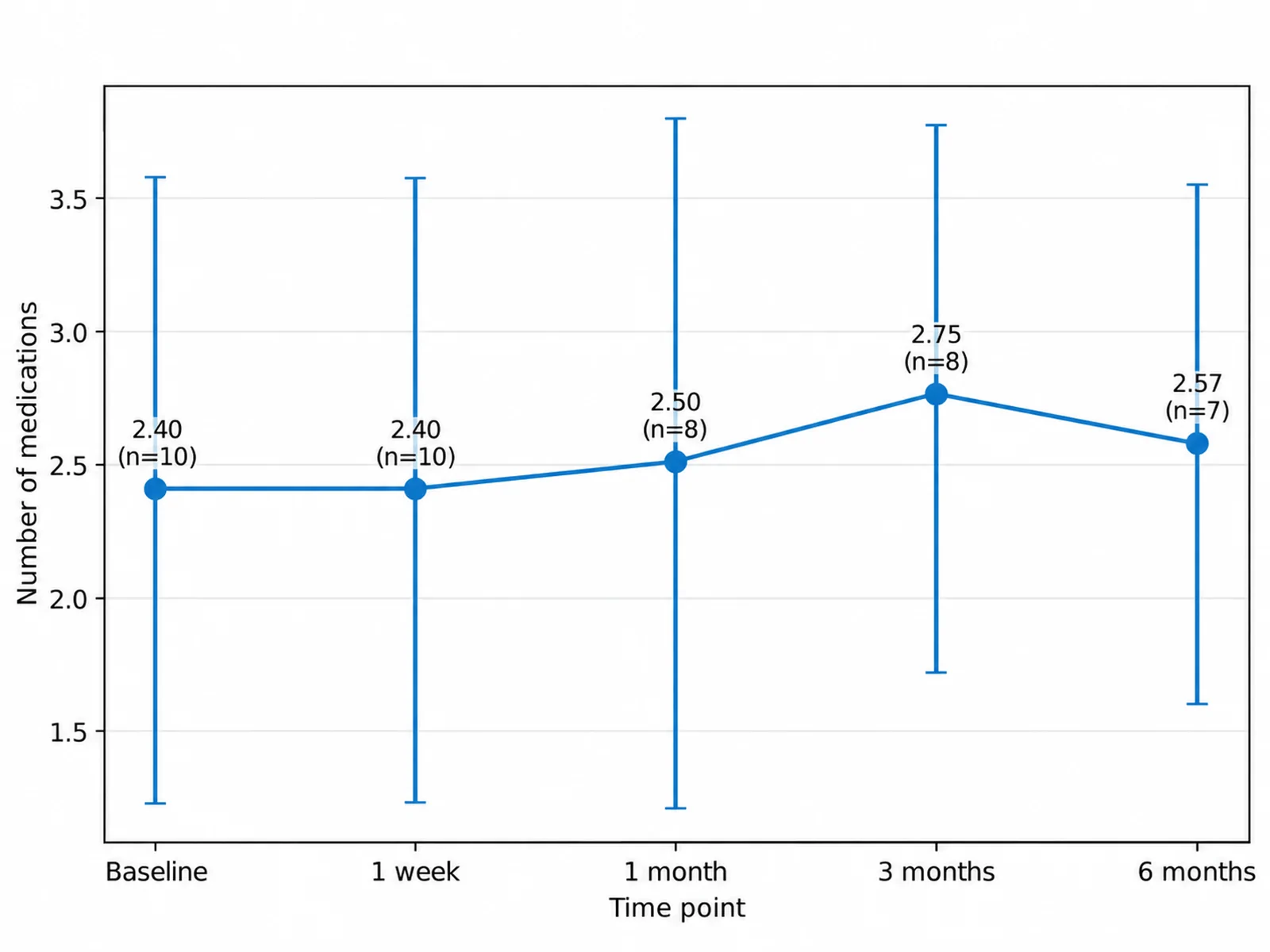
Mean number of antiglaucoma medications across follow-up Mean number of topical antiglaucoma medications at baseline, one week, one month, three months, and six months following selective laser trabeculoplasty (SLT). Error bars represent ±1 standard deviation (SD). The sample size (n) represents the number of treated eyes with available medication data at each time point, decreasing from 10 eyes at baseline to 7 eyes at six months.

Target IOP and subsequent interventions

None of the seven eyes with evaluable six-month data (0/7, 0%) achieved the predefined composite target IOP. Three of the 10 treated eyes (3/10, 30%) underwent subsequent glaucoma surgery: one underwent trabeculectomy before the six-month assessment, while one underwent trabeculectomy and one underwent iStent implantation after completing the six-month follow-up. Among the remaining five eyes that completed the six-month follow-up without subsequent surgery, one had been counselled for trabeculectomy but had not yet decided to proceed.

Follow-up completeness and sensitivity analysis

IOP measurements were available for all 10 eyes (10/10, 100%) at baseline and one week, 8 eyes (8/10, 80%) at one and three months, and 7 eyes (7/10, 70%) at six months. The availability of longitudinal IOP measurements is illustrated in Figure 3.

**Figure 3:**
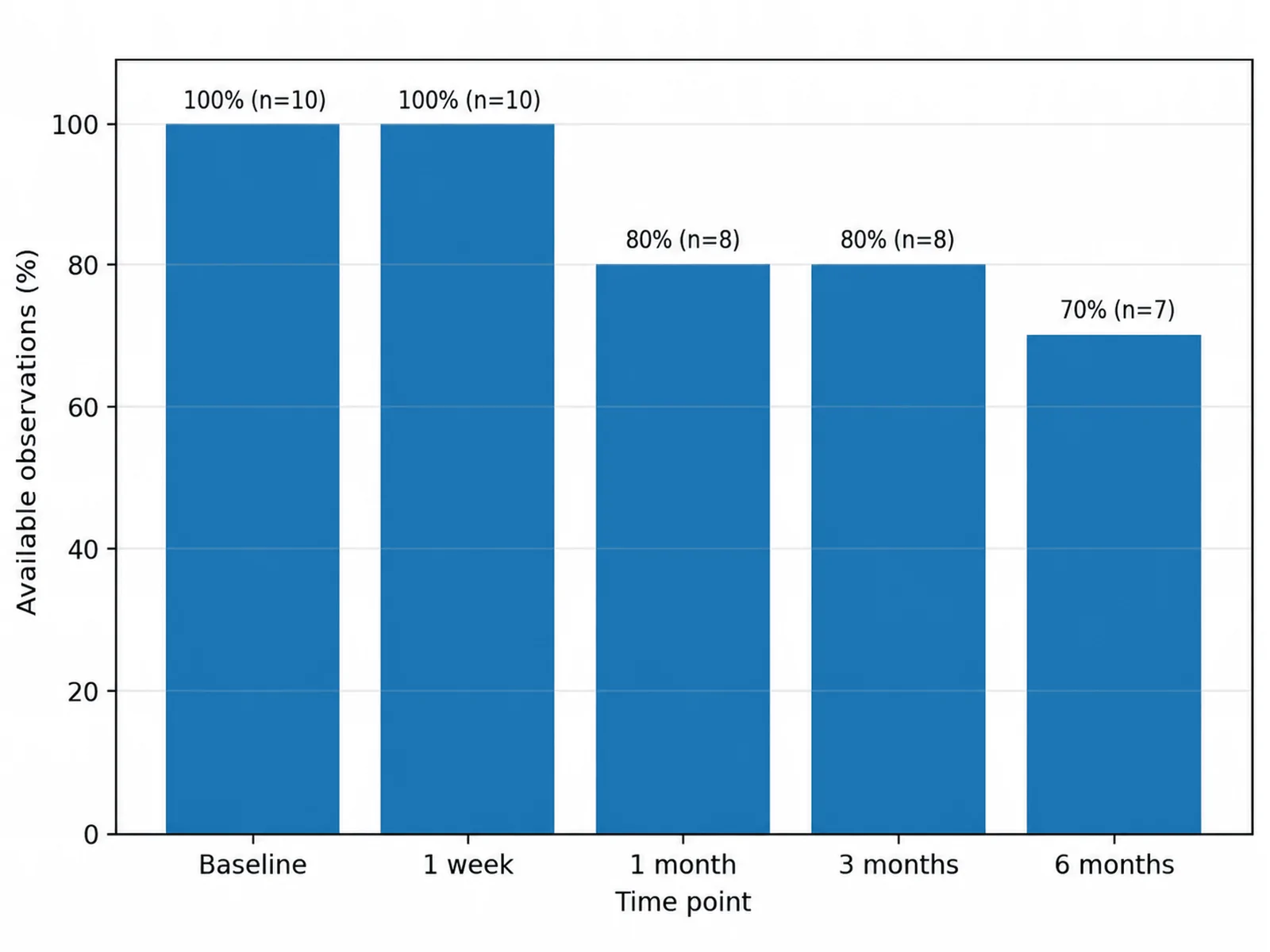
Percentage of eyes with available IOP measurements at each time point The figure illustrates follow-up completeness at baseline (10/10, 100%), one week (10/10, 100%), one month (8/10, 80%), three months (8/10, 80%), and six months (7/10, 70%). Percentages were calculated using the total of 10 treated eyes. IOP: intraocular pressure, n: number of eyes with available measurements.

To account for potential correlation between fellow eyes, a patient-level sensitivity analysis was performed by averaging bilateral IOP measurements within each patient. Five patients had complete measurements across all five time points. The patient-level Friedman test demonstrated no statistically significant change in IOP over time (χ²(4, N = 5) = 2.562, p = 0.634), consistent with the findings of the primary eye-level analysis.

## Discussion

This study evaluated six-month outcomes following VISULAS green SLT in patients with advanced-to-severe POAG. In this small retrospective cohort, improvement in IOP was not demonstrated over six months, and topical IOP-lowering medication burden did not change significantly. Among the seven eyes with complete six-month follow-up, mean IOP increased slightly from 18.86 mmHg at baseline to 19.29 mmHg at six months, representing a paired mean increase of 0.43 mmHg. None of the seven evaluable eyes achieved the predefined composite target IOP. Three of the 10 treated eyes subsequently underwent glaucoma surgery. These findings should be interpreted cautiously because of the very small sample size, incomplete follow-up, and retrospective study design.

Previous studies have demonstrated clinically meaningful IOP reduction following SLT. Pillunat et al. reported an approximately 16% reduction in IOP at one and three months following treatment with VISULAS green SLT [8]. However, their study population differed substantially from the present cohort, which consisted exclusively of patients with advanced-to-severe POAG. Differences in baseline IOP, previous treatment exposure, glaucoma severity, and sample size may have contributed to the differing outcomes observed.

Baseline IOP is recognized as an important determinant of the magnitude of pressure reduction following SLT. Hodge et al. identified higher baseline IOP as a significant predictor of SLT success at one year [12]. Similarly, Pillunat et al. reported that pretreatment IOP was an important predictor of both absolute and relative IOP reduction following SLT in patients already receiving maximally tolerated medical therapy [13]. These observations suggest that glaucoma severity alone may not account for differences in SLT response and that baseline pressure and previous treatment status should also be considered when interpreting outcomes.

An important feature of the present study was the difference between the overall cross-sectional IOP values and the paired complete-case findings. The overall mean IOP numerically decreased from 22.40 ± 7.21 mmHg at baseline to 19.29 ± 2.81 mmHg at six months. However, the number and composition of eyes contributing data changed during follow-up, with only 7 of the original 10 eyes contributing six-month IOP measurements. Among these seven eyes, mean IOP instead increased slightly from 18.86 mmHg at baseline to 19.29 mmHg at six months. This difference illustrates how attrition and changing sample composition can influence cross-sectional summary statistics in small longitudinal studies. The paired findings therefore provide a more appropriate representation of six-month IOP change among eyes with available follow-up.

Evidence specifically evaluating SLT in advanced glaucoma remains comparatively limited. Schlote and Kynigopoulos evaluated SLT in early and advanced open-angle glaucoma and reported variable treatment responses, with some eyes subsequently requiring filtering surgery [11]. This is clinically relevant to the present cohort. One eye underwent trabeculectomy before the six-month assessment, while a further trabeculectomy and one iStent implantation were performed after completion of six-month follow-up. In addition, one eye that completed follow-up had been counselled for trabeculectomy but had not yet decided to proceed. These observations indicate that SLT did not eliminate the need for subsequent treatment escalation in all patients with advanced disease.

Adequate pressure control is particularly important in advanced-to-severe glaucoma because substantial optic nerve damage has already occurred and the remaining visual reserve may be limited. The Malaysian Clinical Practice Guidelines for the Management of Glaucoma emphasize that target IOP should be individualized according to baseline pressure, disease severity, risk of progression, and other patient-specific factors [4]. In the present study, none of the seven eyes with evaluable six-month data achieved the predefined composite target of an IOP of 14 mmHg or lower together with at least a 30% reduction from baseline. This finding highlights the importance of evaluating treatment response against a clinically relevant target rather than relying solely on changes in mean IOP.

Topical IOP-lowering medication burden also did not change significantly during follow-up (p = 0.092). The median medication burden remained at two active IOP-lowering ingredients throughout the study period. This may reflect the advanced disease status of the cohort, in which topical therapy was continued or adjusted to maintain pressure control rather than reduced after SLT. Long-term topical treatment may also contribute to ocular surface disease and treatment burden, particularly in patients requiring multiple medications [6].

These findings differ from those of the Laser in Glaucoma and Ocular Hypertension (LiGHT) trial, in which SLT used as first-line treatment provided effective IOP control while allowing many patients to remain free from topical medication [10]. Direct comparison should, however, be made cautiously. The LiGHT trial primarily evaluated SLT as an initial treatment strategy, whereas the present study involved patients with established advanced-to-severe POAG who were already receiving topical IOP-lowering therapy. Previous reviews have also described the use of SLT as an adjunct to medical therapy, although treatment response varies according to baseline IOP, disease characteristics, and previous treatment exposure [7,9].

The clinical role of SLT in patients with advanced glaucoma should therefore be considered carefully. In selected patients who decline incisional surgery or in whom surgery must be temporarily deferred, SLT may be considered as an adjunctive or interim intervention. However, the findings of the present study do not establish the effectiveness of such an approach, and SLT should not delay definitive surgical treatment when adequate IOP control is not achieved. Close follow-up and timely escalation of treatment remain particularly important in patients with advanced or severe glaucomatous damage.

This study has several important limitations. The very small sample size substantially limited statistical power and the precision of the estimates. The retrospective design introduces the possibility of selection bias and limits control over potentially important confounding factors. Incomplete follow-up may have introduced attrition bias, particularly because the eyes remaining at six months differed from the original baseline cohort. The absence of a control group also prevents attribution of observed IOP changes specifically to SLT. In addition, some patients contributed both eyes, meaning that eye-level observations were not fully independent, although a patient-level sensitivity analysis produced findings consistent with the primary analysis. Medication adjustments and subsequent glaucoma surgery may also have influenced longitudinal IOP measurements. Consequently, the absence of demonstrated improvement should not be interpreted as definitive evidence that SLT has no treatment effect in advanced to severe POAG. The findings should instead be regarded as exploratory and hypothesis-generating, and larger prospective studies with more complete follow-up are needed to better define the role of VISULAS green SLT in this patient population.

## Conclusions

In this small retrospective cohort of patients with advanced-to-severe POAG, VISULAS green SLT did not demonstrate a statistically significant improvement in IOP or topical medication burden over six months. Among the seven eyes with complete six-month data, mean IOP increased slightly from 18.86 mmHg at baseline to 19.29 mmHg at six months. None of the 7 evaluable eyes achieved the predefined target IOP, and 3 of the 10 treated eyes underwent subsequent glaucoma surgery. These findings should be interpreted cautiously because of the very small sample size and incomplete follow-up.

SLT may still have a role as an adjunctive or interim treatment in carefully selected patients when incisional surgery is declined or temporarily deferred. However, close monitoring, individualized target IOP assessment, and timely treatment escalation remain essential when adequate pressure control is not achieved. Larger prospective studies are needed to better define the effectiveness and long-term outcomes of VISULAS green SLT in this patient population.

## References

[1] American Academy of Ophthalmology, Tanna AP (2026). 2023-2024 Basic and Clinical Science Course, Section 10: Glaucoma. https://books.google.com/books/about/2023_2024_Basic_and_Clinical_Science_Cou.html?id=F3jozwEACAAJ&.

[2] Križaj D (2019). What is glaucoma?. Webvision: The Organization of the Retina and Visual System.

[3] Stein JD, Khawaja AP, Weizer JS (2021). Glaucoma in adults-screening, diagnosis, and management: a review. JAMA.

[4] Ministry of Health Malaysia (2017). Management of Glaucoma (Second Edition). Putrajaya, Malaysia: Ministry of Health Malaysia. Clinical Practice Guidelines. Management of Glaucoma (Second Edition).

[5] Heijl A, Leske MC, Bengtsson B, Hyman L, Bengtsson B, Hussein M (2002). Reduction of intraocular pressure and glaucoma progression: results from the Early Manifest Glaucoma Trial. Arch Ophthalmol.

[6] Li G, Akpek EK, Ahmad S (2022). Glaucoma and ocular surface disease: more than meets the eye. Clin Ophthalmol.

[7] Alon S (2013). Selective laser trabeculoplasty: a clinical review. J Curr Glaucoma Pract.

[8] Pillunat KR, Kretz FT, Koinzer S, Ehlken C, Pillunat LE, Klabe K (2023). Effectiveness and safety of VISULAS(®) green selective laser trabeculoplasty: a prospective, interventional multicenter clinical investigation. Int Ophthalmol.

[9] Landers J (2021). Selective laser trabeculoplasty: a review. Clin Exp Ophthalmol.

[10] Gazzard G, Konstantakopoulou E, Garway-Heath D (2019). Selective laser trabeculoplasty versus eye drops for first-line treatment of ocular hypertension and glaucoma (LiGHT): a multicentre randomised controlled trial. Lancet.

[11] Schlote T, Kynigopoulos M (2016). Selective laser trabeculoplasty (SLT): 1-year results in early and advanced open angle glaucoma. Int Ophthalmol.

[12] Hodge WG, Damji KF, Rock W, Buhrmann R, Bovell AM, Pan Y (2005). Baseline IOP predicts selective laser trabeculoplasty success at 1 year post-treatment: results from a randomised clinical trial. Br J Ophthalmol.

[13] Pillunat KR, Spoerl E, Elfes G, Pillunat LE (2016). Preoperative intraocular pressure as a predictor of selective laser trabeculoplasty efficacy. Acta Ophthalmol.

